# Reliability and validity of the Japanese version of the Sleep Problem Acceptance Questionnaire

**DOI:** 10.1371/journal.pone.0358990

**Published:** 2026-10-16

**Authors:** Mizuki Bando, Tomohiro Asakura, Hiroshi Sato, Isa Okajima

**Affiliations:** 1 Graduate School of Humanities and Life Sciences, Tokyo Kasei University, Tokyo, Japan; 2 Graduate School of Humanities, Kwansei Gakuin University, Nishinomiya, Japan; 3 School of Humanities, Kwansei Gakuin University, Nishinomiya, Japan; 4 Behavioral Sleep Medicine and Sciences Laboratory, Department of Psychological Counseling, Faculty of Humanities, Tokyo Kasei University, Tokyo, Japan; Portugal Football School, Portuguese Football Federation, PORTUGAL

## Abstract

This study aimed to develop a Japanese version of the Sleep Problem Acceptance Questionnaire (SPAQ-J) and assess its reliability and validity. The SPAQ-J was developed through a forward-backward translation procedure with permission from the original authors. The sample included 861 adults with insomnia who completed questionnaires on experiential avoidance, insomnia symptoms, dysfunctional beliefs about sleep, sleep-related safety behaviors, insomnia catastrophizing, and pre-sleep arousal. Confirmatory factor analysis confirmed that the SPAQ-J retained the two-factor structure of the original version (CFI = 0.972, TLI = 0.958, RMSEA = 0.082, SRMR = 0.059): Activity Engagement and Willingness. Although internal consistency was adequate (Activity Engagement: α = 0.868, ω = 0.870; Willingness: α = 0.895, ω = 0.895), test-retest reliability was below the recommended threshold (Activity Engagement: ICC = 0.586, Willingness: ICC = 0.589). In terms of construct validity, Willingness moderately correlated with all scales, whereas Activity Engagement showed moderate-to-weak correlations. Hierarchical multiple regression analysis revealed that adding the SPAQ-J subscales significantly enhanced the explanatory power. Measurement invariance analysis across sex and age groups showed no significant changes in the fit indices, indicating stability. These findings suggest that the SPAQ-J is a valid and partially reliable measure of psychological flexibility related to sleep problems in the Japanese population.

## Introduction

Insomnia is a major global public health concern. Insomnia is characterized by symptoms such as difficulty falling asleep, difficulty staying asleep, or early morning awakening, often accompanied by daytime impairments such as sleepiness or fatigue [1]. The global prevalence of insomnia is 16.2% [2], and insomnia is associated with increased suicide risk [3], heightened cardiovascular disease risk [4], and reduced quality of life [5]. Various factors contribute to insomnia, with sex and age being significant [6]. A systematic review indicated a higher prevalence in women across all age groups, particularly in those aged 35–50 years [2]. Thus, insomnia affects a large proportion of the population across their lifespan, prompting the development of psychological interventions.

Cognitive behavioral therapy for insomnia (CBT-I) is a highly successful first-line intervention for insomnia disorder [7]. However, the effect of CBT-I on daytime symptoms is weaker than on nocturnal symptoms [8]. This weak point has led to the proposal of various interventions aiming to comprehensively address daytime symptoms as well [9]. Among these, Acceptance and Commitment Therapy (ACT [10]) has attracted growing attention in recent years. ACT encourages individuals to relinquish their attempts to control sleep, facilitate natural sleep onset, and promote daytime activities, such as value-aligned actions, to enhance their quality of life beyond merely alleviating insomnia [11]. It can be used as a standalone treatment or in conjunction with CBT-I techniques, such as stimulus control and sleep restriction, both of which have shown effectiveness in improving insomnia symptoms [12]. Additionally, ACT has been applied to improve sleep quality in non-clinical populations [13,14], with growing research on its efficacy and mechanisms as a novel intervention for insomnia.

A key focus of ACT for insomnia is psychological flexibility, which is defined as the capacity to accept internal experiences such as unpleasant emotions and thoughts while choosing value-consistent actions [10]. Research indicates that psychological inflexibility is a significant predictor of insomnia severity and daytime symptoms, such as obsessive-compulsive symptoms and pain, in both clinical and non-clinical populations [15], and that all six core processes of flexibility may correlate with insomnia severity and sleep quality [16]. Therefore, an accurate assessment of psychological flexibility in sleep research is crucial.

The Acceptance and Action Questionnaire-II (AAQ-II [17]) is the most widely used measure of psychological inflexibility, serving as a process indicator for ACT interventions and for assessing general psychological flexibility and experiential avoidance [18]. In previous studies, the AAQ-II was used to gauge ACT-based improvements; however, these studies have produced inconsistent results regarding the connection between changes in psychological flexibility and insomnia symptoms. Some studies found improved AAQ-II scores without corresponding reductions in insomnia severity [19], whereas others noted insomnia improvements without changes in AAQ-II scores [20]. The lack of consistent findings may stem from the general design of the AAQ-II, which may not effectively capture psychological flexibility changes specific to sleep problems. Therefore, a measure of psychological flexibility specific to sleep problems would be beneficial for evaluating insomnia.

The Sleep Problem Acceptance Questionnaire (SPAQ), developed by Bothelius et al. [21], is a valid tool for assessing psychological flexibility in sleep disorders. Adapted from the Chronic Pain Acceptance Questionnaire (CPAQ [22]), the SPAQ measures acceptance of sleep issues through two factors: activity engagement, which evaluates the willingness to continue normal activities despite perceived insufficient sleep; and willingness, which assesses the acceptance of sleep problems without resistance. Both factors have demonstrated high reliability and validity, showing stronger associations with insomnia symptoms than the Dysfunctional Beliefs and Attitudes about Sleep Scale (DBAS [23]) or Sleep-related Behaviors Questionnaire (SRBQ [24]) [21].

Sleep-specific measures of psychological flexibility are currently limited to the SPAQ and the Sleep Acceptance Scale (SAS) [25]. Of them, the SPAQ is the only measure that has been adopted in multiple ACT intervention studies for insomnia [26,27]. However, besides the original version, the SPAQ has only been translated and validated in a Brazilian Portuguese version [28], and no validated Japanese version is available. Previous studies in Japan have reported associations between psychological flexibility and insomnia severity [29], highlighting the importance of assessing this construct in the context of sleep. A validated Japanese version of the SPAQ would facilitate future ACT for insomnia research and clinical evaluation in Japan while enabling findings from Japanese studies to be interpreted alongside the international literature.

This study aimed to develop a Japanese version of the SPAQ and assess its reliability and validity, focusing on internal consistency and test-retest reliability, construct validity, and measurement invariance by sex and age.

We also evaluated the validity of total SPAQ scores. Although the original [21] and Brazilian versions [28] emphasize the assessment of each subscale, the total score has been used in ACT interventions for insomnia [27]. Concerns about its Cronbach’s α (α = 0.59) [21] and the negative correlation between the two subscales [21,28] challenge the validity of treating it as a single construct. Therefore, we explored the factor structure of the Japanese version of the SPAQ using one-factor, two-factor, and bifactor models to better understand its applicability to total scores. The following hypotheses were examined in this study:

### Hypotheses

#### Structural validity verification.

H1. The SPAQ-J would retain the original two-factor structure of the SPAQ. Confirmatory factor analysis (CFA) was conducted using the original two-factor model from the SPAQ [21]. Model fit was considered adequate according to the criteria proposed by Hu and Bentler [30]: comparative fit index (CFI) ≥ 0.900, Tucker–Lewis index (TLI) ≥ 0.900, standardized root mean square residual (SRMR) ≤ 0.08, and root mean square error of approximation (RMSEA) ≤ 0.100, as well as sufficient factor loadings (λ ≥ 0.400 [31]). Additionally, to assess the usability of the total SPAQ score, we performed CFA using one-, two-, or bifactor models.

#### Reliability verification.

H2. The SPAQ-J would demonstrate satisfactory internal consistency and test-retest reliability. Internal consistency was evaluated using Cronbach’s α and McDonald’s ω at the first measurement point, with satisfactory values defined as α ≥ 0.700, ω ≥ 0.700 [32]. Test-retest reliability was assessed via the intraclass correlation coefficient (ICC) using a two-way mixed model for absolute agreement for SPAQ item groups, with sufficient values defined as ICC ≥ 0.700 [33].

#### Construct validity verification.

H3. The SPAQ-J would show moderate associations with theoretically related constructs, supporting convergent validity. For convergent validity, we calculated 95% confidence intervals (CIs) for correlation coefficients between the SPAQ item groups and other validated scales, including experiential avoidance, insomnia symptoms, dysfunctional sleep beliefs, sleep-related safety behaviors, insomnia catastrophizing, and pre-sleep arousal [21]. Because higher SPAQ-J scores indicate greater psychological flexibility, whereas higher scores on the comparison measures indicate greater difficulties, all correlations were expected to be negative in direction. We considered the association to be moderate when the absolute value of the 95% CI for the correlation coefficient was 0.300 or greater [34].

H4. The SPAQ-J factors would explain additional variance in insomnia symptoms beyond dysfunctional sleep beliefs and sleep-related safety behaviors, supporting incremental validity. For incremental validity, we conducted hierarchical multiple regression analysis with insomnia symptoms as the dependent variable. Step 1 included dysfunctional sleep beliefs and sleep-related safety behaviors, and Step 2 added the two SPAQ factors.

#### Verification of measurement invariance.

H5. The SPAQ-J would demonstrate measurement invariance across sex and age groups. We performed a simultaneous multi-group analysis across sex and age groups to examine measurement invariance, testing whether differences between the configural invariance model and the metric, scalar, and strict invariance models did not exceed established reference criteria (ΔCFI ≤ 0.010, ΔSRMR ≤ 0.030, ΔRMSEA ≤ 0.015 [35]).

## Methods

### Research participants and procedures

Participants were recruited from an online survey panel managed by Cross Marketing, Inc. which consisted of individuals aged 20–69 years. Cross Marketing, Inc. had 13.22 million active panelists in the past year. The study focused on individuals with insomnia disorder, as defined by the American Psychiatric Association criteria [1]: difficulty falling asleep or staying asleep for 30 min or longer, or early morning awakenings occurring three or more days per week, persisting for at least three months. Additionally, daytime functional impairment was assessed through difficulties in daily life caused by nocturnal symptoms such as increased fatigue, decreased mood, and reduced activity levels. Individuals receiving pharmacological or psychological treatment for physical or mental disorders were excluded.

To ensure that the respondents read the questionnaire thoroughly, two satisficing items were employed [36], and those who answered incorrectly were excluded from the analysis. The surveys were conducted on November 18, 2025 (Time 1) and December 8, 2025 (Time 2).

The survey company distributed the online survey to 409,232 registered panelists. After excluding individuals who did not meet the eligibility criteria based on the screening items or did not complete the survey, 1,422 participants completed the survey. Of them, 561 were excluded due to incomplete or invalid responses, resulting in 861 participants at Time 1. At Time 2 (two weeks later), 490 participants were excluded due to non-response or invalid responses, resulting in 371 participants at Time 2. The participant recruitment process is illustrated in Fig 1.

**Fig 1:**
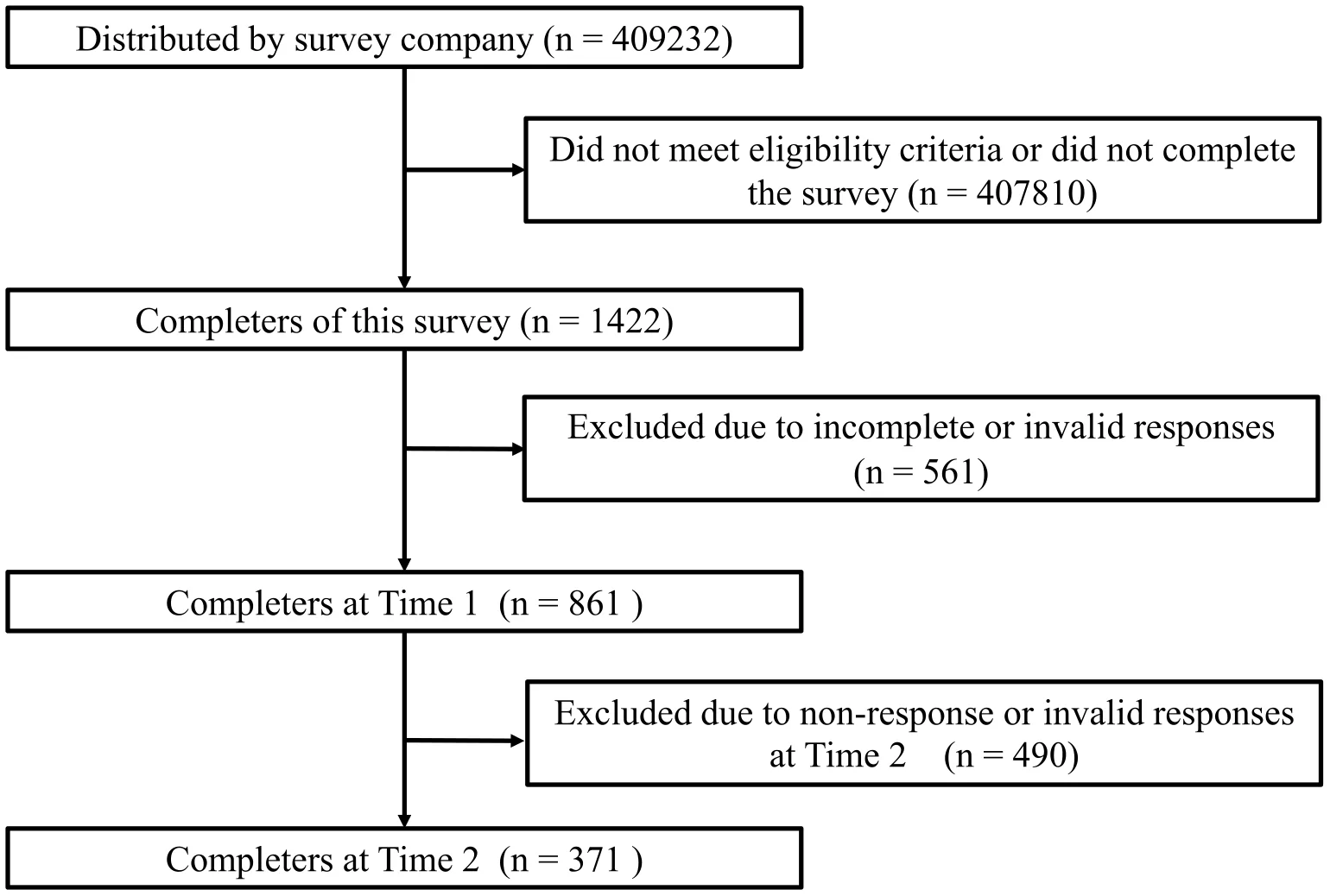
Flowchart of the participant selection for this study.

### Research materials

#### Demographic items.

Participants provided information on sex, age, and occupation.

#### Screening items.

Participants reported nocturnal insomnia symptoms (difficulty falling asleep, difficulty staying asleep, and early morning awakening) and daytime functional impairments.

#### SPAQ.

The original version included two factors—Activity Engagement and Willingness—and eight items. Responses were measured on a 7-point scale, with each subscale scoring from 0 to 24 points, and a total score from 0 to 48 points. The four reverse-scored items of willingness were adjusted in the analysis, such that higher scores indicated greater psychological flexibility regarding sleep problems.

The SPAQ [21] was translated with permission from the original authors. First, we engaged an academic translation service for forward and reverse translations of the instructions and items, resulting in a provisional Japanese version. The back translation was then reviewed by the original author, who provided feedback. The second author, a doctoral clinical psychology student, and third author, a university faculty member with experience in scale translation, refined the expressions. The fourth author reviewed the appropriateness and expression issues. The third author, an expert in CBT with a PhD in clinical psychology, has six years of experience as the editor-in-chief of the Japanese Journal of Behavioral and Cognitive Therapies. The fourth author, also a PhD. holder in clinical psychology, specializes in clinical behavior analysis and CBT, with eight years as editor-in-chief and deputy editor-in-chief of the same journal, alongside 18 years of CBT and ACT experience. To revise the forward and back translations, we used a translation service. After five rounds of linguistic equivalence verification through back-translation, the necessary revisions were made. The original author confirmed the linguistic equivalence. Subsequently, cognitive debriefing was conducted with several Japanese adults, who individually completed the translated items and provided verbal feedback on item clarity and naturalness of the wording. This process confirmed that the item content posed no issues. However, pilot testing and interviews with individuals with insomnia were not conducted prior to the main survey. This led to the creation of the final version.

#### AAQ-II.

The AAQ-II, developed by Bond et al. [17] and adapted into Japanese by Shima et al. [37], assesses experiential avoidance with seven items on a 7-point scale ranging from “Strongly disagree: 1” to “Always: 7,” yielding scores from 7 to 49. Its reliability and validity were confirmed by Shima et al. [37], with higher scores indicating greater experiential avoidance.

#### Athens Insomnia Scale (AIS).

The AIS, created by Soldatos et al. [38] and adapted into Japanese by Okajima et al. [39], measures insomnia symptoms using two factors and eight items: five (AIS-N) assess nocturnal symptoms, and three (AIS-D) assess daytime functional impairment. Each item is rated on a 4-point scale, with total scores ranging from 0 to 24. Okajima et al. [39] have confirmed their reliability and validity. Higher scores indicate more severe insomnia symptoms.

The original study [21] used the Insomnia Severity Index (ISI) to measure insomnia symptoms, whereas this study used the AIS. Unlike the ISI, the AIS consistently supports a two-factor structure of nighttime symptoms and daytime functioning [38,39]. The SPAQ includes items on both daytime and nighttime sleep issues, making the AIS a suitable indicator for assessing insomnia symptoms separately for day and night. Both the ISI and AIS have equivalent sensitivity and specificity for insomnia disorder [40] and can be classified into four categories: no insomnia, mild, moderate, and severe [41]. Therefore, this study adopted the AIS for comparison with the original research.

#### Dysfunctional Beliefs and Attitudes about Sleep Scale-16 (DBAS).

The DBAS, created by Morin et al. [42] and adapted into Japanese by Munezawa et al. [43], measures dysfunctional beliefs about sleep using 16 items across four factors. Responses are provided on an 11-point scale: “Not at all applicable: 0” to “Strongly agree: 10,” with scores ranging from 0 to 160. Munezawa et al. [43] reported its reliability and validity. Higher scores indicated stronger dysfunctional beliefs about sleep.

#### Short version of SRBQ.

The Sleep-Related Behaviours Questionnaire (SRBQ), created by Ree and Harvey [24] and adapted into Japanese by Ubara et al. [44], assesses safety behaviors related to sleep using one factor and 15 items. Responses are given on a 5-point scale: “Not at all: 0” to “Always: 4,” with possible scores from 0 to 60. Ubara et al. [44] documented its reliability and validity. Higher scores indicate more safety behaviors related to sleep.

#### Insomnia Catastrophizing Scale (ICS).

Created by Jansson-Fröjmark et al. [45] and adapted into Japanese by Takano et al. [46], this scale measured catastrophic thinking about insomnia. It includes two factors and 17 items: 11 for nighttime thinking (ICS-N) and six for daytime thinking (ICS-D). Responses are rated on a 6-point scale: “Not at all: 0” to “Always: 5.” The score range for nighttime thinking was 0–55 points, while that for daytime thinking was 0–30 points. Takano et al. [46] reported their reliability and validity. Higher scores indicated stronger catastrophizing.

#### Pre-Sleep Arousal Scale (PSAS).

Developed by Nicassio et al. [47] and adapted for Japanese by Okajima et al. [48], this scale measures pre-sleep hyperarousal using 16 items divided into two factors: the 8-item Physical Hyperarousal Scale (PSAS-S) and the 8-item Cognitive Hyperarousal Scale (PSAS-C). Participants respond on a 5-point scale from “Never: 1” to “Very often: 5,” with scores ranging from 8 to 40 for both physical and cognitive hyperarousal. Okajima et al. [48] established the reliability and validity of this scale. Higher scores indicate greater hyperarousal before the onset of sleep.

### Ethical considerations

All procedures were performed in accordance with the ethical standards of the Institutional Research Committee and relevant guidelines and regulations, including the Declaration of Helsinki. This study was approved by the Research Ethics Committee of Tokyo Kasei University (approval number: R7-17; approval date: October 2, 2025). The web survey stated four key points: (a) participation was voluntary and responses were not mandatory; (b) responses were anonymous, with personal information kept confidential; (c) data were used exclusively for research purposes; and (d) there were no correct or incorrect answers. Participants who completed the questionnaire were considered to have provided consent to participate in the study.

### Data analysis

Statistical analyses were performed using R version 4.5.2 (R Foundation, Vienna, Austria). The dataset delivered by the survey company contained no missing values, as responses with incomplete or missing items had already been excluded prior to delivery. Descriptive statistics, including means, standard deviations, ranges, skewness, and kurtosis, were calculated using the psych package (version 2.6.3 [49]). For sample sizes greater than 300, absolute skewness values below 2 and absolute kurtosis values below 7 were considered indicative of an approximately normal distribution [50]. Data and analysis code are available at: https://osf.io/8cgdb/.

#### Structural validity.

To assess the structural validity of the Japanese version of the SPAQ, CFA was conducted using the maximum likelihood estimator with the lavaan package (version 0.6.21) [51,52]. Although full information maximum likelihood (FIML) was specified in the analysis code, the dataset contained no missing values; therefore, FIML yielded the same estimates as standard maximum likelihood estimation. In addition to the previously confirmed two-factor model, we tested both one-factor and bifactor models. Adequate fit indices were defined as CFI ≥ 0.900, TLI ≥ 0.900, SRMR ≤ 0.080, and RMSEA ≤ 0.100 [30], with λ ≥ 0.400 considered a sufficient factor loading value [31].

#### Reliability.

Floor and ceiling effects were examined for the SPAQ item groups by calculating the percentage of participants who obtained the lowest or highest possible score. Floor or ceiling effects were considered present if more than 15% of participants achieved the lowest or highest possible score, respectively [53]. Internal consistency was evaluated using Cronbach’s α and McDonald’s ω coefficients, calculated with the psych package (version 2.6.3 [49]) and the semTools package (version 0.5.7 [54]), respectively. Values of α ≥ 0.700 and ω ≥ 0.700 were considered sufficient [32]. Test-retest reliability was assessed using the psych package (version 2.6.3 [49]) to calculate the ICC via a two-way mixed model for absolute agreement, with ICC ≥ 0.700 indicating satisfactory reliability [33].

#### Construct validity.

Construct validity was assessed using the psych package (version 2.6.3 [49]) to compute Pearson’s product-moment correlation coefficients and 95% CIs between the SPAQ item groups and other validated scales, including insomnia symptoms, experiential avoidance, dysfunctional beliefs, safety behaviors, nighttime/daytime catastrophic thinking, and physical/cognitive hyperarousal. Linearity was evaluated by visual inspection of scatterplots for each variable pair prior to the correlation analyses. We determined a moderate or stronger association was present if the absolute value of the 95% CI for the correlation coefficient was 0.300 or greater, based on Cohen’s [34] criterion of *r* = 0.300.

Additionally, hierarchical multiple regression analysis with forced insertion was performed to assess the incremental validity of the SPAQ in predicting insomnia symptoms. We evaluated whether including AIS as the dependent variable and DBAS and SRBQ as independent variables in Step 1, along with Activity Engagement and Willingness subscales for SPAQ in Step 2, improved the relationship with AIS and increased explanatory power. Standardized regression coefficients were calculated using the lm.beta package (version 1.7.3 [55]), and 95% CIs were estimated using the Bias-Corrected and Accelerated method (bootstrap replications: 2000) implemented in the boot package (version 1.3–32 [56,57]). To determine the unique contribution of each predictor variable to the outcome variance in Step 2, we calculated the semi-partial correlation (*sr*) and its squared value (*sr^2^*). The 95% CI for *sr* was calculated using the Bias-Corrected and Accelerated method (bootstrap replications: 2000). Prior to the regression analyses, residual normality was evaluated using Q-Q plots. Linearity and homoscedasticity were assessed by visual inspection of residual-versus-fitted plots, and homoscedasticity was additionally evaluated using the Breusch-Pagan test [58]. Multicollinearity was assessed using variance inflation factors (VIFs), with values below 5 considered acceptable [59]. When the Breusch-Pagan test indicated heteroscedasticity, HC3 robust standard errors were additionally applied as a sensitivity analysis to account for potential heteroscedasticity [60].

#### Measurement invariance.

Measurement invariance was assessed through simultaneous multi-population analyses of sex and age groups. Because of the small number of respondents who declined to answer, analyses were conducted separately for men and women. The age-based analysis divided the participants into three groups: young (20–35 years), middle-aged (36–50 years), and older adults (51–69 years). We used the lavaan package (version 0.6.21 [51,52]) to conduct multi-group confirmatory factor analysis. First, we established a model that assumes factor structure invariance (configural invariance). Next, we developed a metric invariance model with constraints on factor loadings, followed by a scalar invariance model with additional constraints on item intercepts, and finally a strict invariance model with additional constraints on residual variances. Fit indices were calculated for each model, and changes were assessed based on Chen’s criteria (ΔCFI ≤ 0.010, ΔSRMR ≤ 0.030, ΔRMSEA ≤ 0.015) [35].

## Results

### Summary statistics

The demographic information of the participants is shown in Table 1, and summary statistics are shown in Table 2. All variables met the predefined criteria for approximate normality.

**Table 1:** Sociodemographic data of participants.

	Time 1	Time 2
Age, *n* (%)		
20-35	229 (26.6)	101 (27.2)
36-50	289 (33.6)	125 (33.7)
51-69	343 (39.8)	145 (39.1)
mean (*SD*)	45.42 (13.52)	45.49 (13.36)
Sex, *n* (%)		
Male	403 (46.8)	176 (47.4)
Female	451 (52.4)	190 (51.2)
Prefer not to answer	7 (0.8)	5 (1.3)
Insomnia severity (AIS score), n (%)		
Mild or below (≤ 9)	285 (33.1)	131 (35.3)
Moderate (10–15)	413 (48.0)	168 (45.3)
Severe (≥16)	163 (18.9)	72 (19.4)
Occupation, *n* (%)		
Full-time employed	462 (53.7)	206 (55.5)
Part-time employed	133 (15.4)	52 (14.0)
Student	27 (3.1)	8 (2.2)
Homemaker	86 (10.0)	31 (8.4)
Unemployed	125 (14.5)	63 (17.0)
Other	28 (3.3)	11 (3.0)

*Note*. AIS, Athens Insomnia Scale

**Table 2:** Descriptive statistics for each scale.

	Time 1 (*N* = 861)					Time 2 (*N* = 371)				
	*Mean*	*SD*	*Min-Max*	*Skewness*	*Kurtosis*	*Mean*	*SD*	*Min-Max*	*Skewness*	*Kurtosis*
Activity Engagement	10.76	5.46	0-24	.17	−.39	10.28	5.61	0-24	.02	−.66
Willingness	12.19	5.45	0-24	−.19	−.50	12.22	5.43	0-24	−.25	−.37
AAQ-II	29.10	9.81	7-49	.04	−.44	28.78	10.33	7-49	.01	−.49
AIS	11.96	4.33	0-24	.68	.30	11.64	4.70	0-24	.63	.21
AIS-N	7.64	2.82	0-15	.59	.25	7.50	3.03	0-15	.67	.26
AIS-D	4.32	1.99	0-9	.49	−.19	4.14	2.11	0-9	.57	−.03
DBAS	92.31	23.85	9-160	−.07	.50	92.12	25.81	0-160	−.22	.40
SRBQ	26.66	11.91	0-60	.18	−.27	26.77	12.94	0-60	.23	−.35
ICS-N	31.84	11.12	0-55	−.21	.04	31.66	12.13	0-55	−.25	−.20
ICS-D	16.20	7.42	0-30	−.06	−.59	16.13	7.84	0-30	−.17	−.63
PSAS-S	18.56	7.45	8-40	.69	−.20	18.66	7.64	8-40	.65	−.38
PSAS-C	24.18	7.83	8-40	−.03	−.75	24.31	8.39	8-40	.01	−.92

*Note*. AAQ-II, Acceptance and Action Questionnaire-II; AIS, Athens Insomnia Scale; AIS-N, Athens Insomnia Scale-Nocturnal; AIS-D, Athens Insomnia Scale-Daytime; DBAS, Dysfunctional Beliefs and Attitudes about Sleep-16; SRBQ, Sleep-related Behaviors Questionnaire; ICS-N, Insomnia Catastrophizing Scale-Nighttime; ICS-D, Insomnia Catastrophizing Scale-Daytime; PSAS-S, Pre-Sleep Arousal scale-somatic; PSAS-C, Pre-Sleep arousal scale-cognitive.

### Confirmatory factor analysis

We performed a CFA based on a two-factor model from a previous study [21]. The analysis indicated an acceptable model fit (χ^2^(19) = 127.826, *p* < 0.001, CFI = 0.972, TLI = 0.958, RMSEA [90% CI] = 0.082 [0.068, 0.095], SRMR = 0.059, Akaike information criterion (AIC) = 22199.452, Bayesian information criterion (BIC) = 22318.404). The correlation coefficient between the two factors was weak and negative (*r* = −0.171). The factor loadings are shown in Fig 2.

**Fig 2:**
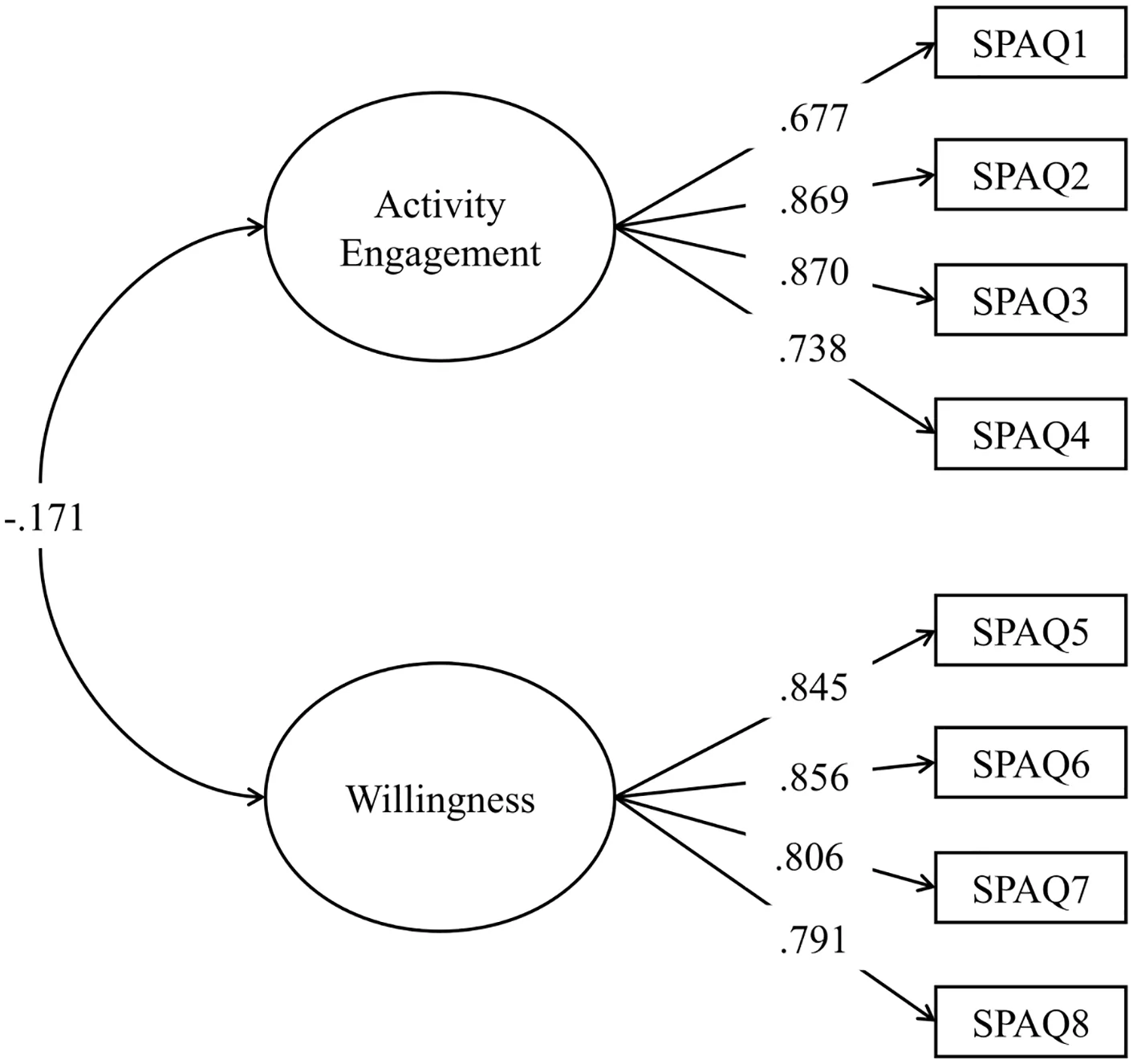
Two-factor model of the SPAQ using confirmatory factorial analysis.

Additionally, we examined the construct validity of the SPAQ total score by conducting CFAs on one-factor and bifactor models (S1 Fig). The one-factor model showed inadequate fit (χ^2^(20) = 2055.420, *p* < 0.001, CFI = 0.471, TLI = 0.259, RMSEA = 0.344 [0.331, 0.356], SRMR = 0.241, AIC = 24125.046, BIC = 24239.240). Noting negative paths in some items, we conducted a bifactor analysis, which demonstrated adequate fit (χ^2^(12) = 18.023, *p* = 0.115, CFI = 0.998, TLI = 0.996, RMSEA = 0.024 [0.000, 0.046], SRMR = 0.012, AIC = 22103.649, BIC = 22255.908). However, the general factor loadings were positive (0.361–0.401) for Activity Engagement items and negative (−0.590 to −0.608) for Willingness items, indicating that the model may not adequately represent the latent constructs. Thus, the construct validity of the total SPAQ score was deemed unsupported, leading to the adoption of a two-factor model and exclusion of the total score from subsequent analyses.

### Reliability

#### Floor and ceiling effects.

Floor and ceiling effects were examined for each subscale. No floor or ceiling effects were observed for either the Activity Engagement or Willingness subscale, with 2.7% and 2.2% of participants scoring at the minimum and maximum possible values for Activity Engagement, respectively, and 2.6% and 1.5% for Willingness.

#### Internal consistency.

Both the Activity Engagement and Willingness subscales demonstrated strong internal consistency, with Cronbach’s α and McDonald’s ω coefficients exceeding the benchmark (Activity Engagement: α = 0.868 [0.853, 0.882], ω = 0.870 [0.849, 0.887]; Willingness: α = 0.895 [0.883, 0.906], ω = 0.895 [0.881, 0.910]).

#### Test-retest reliability.

The ICCs (2,1) for Activity Engagement and Willingness were below the reference ranges: Activity Engagement (ICC = 0.586 [0.515, 0.649]) and Willingness (ICC = 0.589 [0.518, 0.651]).

### Construct validity

Pearson’s correlation coefficients between the variables are shown in Table 3. Visual inspection of scatterplots indicated no substantial deviations from linearity for any variable pair. Correlation coefficients were calculated to assess the convergent validity. Activity Engagement exhibited significant negative correlations exceeding the hypothesized criterion with AAQ-II (*r =* −0.274 [−0.335, −0.211], *p* < 0.001), AIS-D (*r =* −0.254 [−0.316, −0.191], *p* < 0.001), and ICS-D (*r =* −0.314 [−0.373, −0.253], *p* < 0.001). Although correlations with AIS (*r =* −0.210 [−0.273, −0.146], *p* < 0.001), AIS-N (*r =* −0.114 [−0.209, −0.078], *p* < 0.001), DBAS (*r =* −0.163 [−0.227, −0.097], *p* < 0.001), SRBQ (*r =*−0.199 [−0.263, −0.134], *p* < 0.001), ICS-N (*r =* −0.208 [−0.271, −0.143], *p* < 0.001), PSAS-S (*r =* −0.180 [−0.244, −0.115], *p* < 0.001), and PSAS-C (*r =* −0.181 [−0.245, −0.116], *p* < 0.001) were significant, they fell below the hypothesized criterion.

**Table 3:** Correlation coefficients between the SPAQ and each scale.

	SPAQ			
	Pearson’s *r* [95% CI]			
	Activity Engagement		Willingness	
AAQ-II	−.274**	[−.335, −.211]	−.305**	[−.365, −.243]
AIS	−.210**	[−.273, −.146]	−.335**	[−.393, −.275]
AIS-N	−.114**	[−.209, −.078]	−.313**	[−.372, −.251]
AIS-D	−.254**	[−.316, −.191]	−.288**	[−.348, −.225]
DBAS	−.163**	[−.227, −.097]	−.482**	[−.532, −.429]
SRBQ	−.199**	[−.263, −.134]	−.413**	[−.467, −.356]
ICS-N	−.208**	[−.271, −.143]	−.471**	[−.521, −.417]
ICS-D	−.314**	[−.373, −.253]	−.389**	[−.444, −.330]
PSAS-S	−.180**	[−.244, −.115]	−.269**	[−.330, −.206]
PSAS-C	−.181**	[−.245, −.116]	−.333**	[−.391, −.272]

*Note*. AAQ-II, Acceptance and Action Questionnaire-II; AIS, Athens Insomnia Scale; AIS-N, Athens Insomnia Scale-Nocturnal; AIS-D, Athens Insomnia Scale-Daytime; DBAS, Dysfunctional Beliefs and Attitudes about Sleep-16; SRBQ, Sleep-related Behaviors Questionnaire; ICS-N, Insomnia Catastrophizing Scale-Nighttime; ICS-D, Insomnia Catastrophizing Scale-Daytime; PSAS-S, Pre-Sleep Arousal scale-somatic; PSAS-C, Pre-Sleep arousal scale-cognitive **p < 0.001.

Willingness showed significant negative correlations exceeding the hypothesized criterion with AAQ-II (*r =* −0.305 [−0.365, −0.243], *p* < 0.001), AIS (*r =* −0.335 [−0.393, −0.275], *p* < 0.001), AIS-N (*r =* −0.313 [−0.372, −0.251], *p* < 0.001), AIS-D (*r =* −0.288 [−0.348, −0.225], *p* < 0.001), DBAS (*r =* −0.482 [−0.532, −0.429], *p* < 0.001), SRBQ (*r =* −0.413 [−0.467, −0.356], *p* < 0.001), ICS-N (*r =* −0.471 [−0.521, −0.417], *p* < 0.001), ICS-D (*r =* −0.389 [−0.444, −0.330], *p* < 0.001), PSAS-S (*r =* −0.269 [−0.330, −0.206], *p* < 0.001), and PSAS-C (*r =* −0.333 [−0.391, −0.272], *p* < 0.001).

Visual inspection of the Q-Q plots indicated no substantial violations of residual normality. Inspection of the residual-versus-fitted plots also indicated no substantial violations of linearity or homoscedasticity. Although the Breusch-Pagan test indicated mild heteroscedasticity in all three models, sensitivity analyses based on HC3 robust standard errors yielded the same substantive conclusions as the primary analyses. VIF values ranged from 1.19 to 1.89 across all models, indicating no evidence of multicollinearity. Hierarchical multiple regression analysis using forced entry was conducted to assess the incremental validity of the SPAQ in predicting insomnia symptoms (Table 4). The Step 1 model was significant (*R^2^* = 0.305, *p* < 0.001), indicating positive associations between AIS and both DBAS (*β* = 0.279 [0.202, 0.351], *p* < 0.001) and SRBQ (*β* = 0.333 [0.252, 0.413], *p* < 0.001). The Step 2 model showed a significant increase in explanatory power (*R^2^* = 0.329, *ΔR*^2^ = 0.024, *p* < 0.001). DBAS (*β* = 0.217 [0.133, 0.296], *p* < 0.001, *sr* = 0.158 [0.097, 0.218], *sr^2^* = 0.025), SRBQ (*β* = 0.283 [0.195, 0.364], *p* < 0.001, *sr* = 0.213 [0.150, 0.274], *sr^2^* = 0.046), Activity Engagement (*β* = −0.148 [−0.210, −0.081], *p* < 0.001, *sr* = −0.135 [−0.191, −0.075], *sr²* = 0.018), and Willingness (*β* = −0.144 [−0.222, −0.066], *p* < 0.001, *sr* = −0.116 [−0.181, −0.055], *sr²* = 0.014) were significantly associated with AIS.

**Table 4:** Results of the hierarchical regression analyses.

(*n* = 861)		B	SE	β	[95%CI]	s*r*	[95%CI]	s*r*²	*R* ^2^	*ΔR* ^2^
Outcome variable										
AIS-total	Step 1									
	DBAS	.051	.007	.279**	[.202,.351]				.305**	
	SRBQ	.121	.013	.333**	[.252,.413]					
	Step 2									
	DBAS	.039	.007	.217**	[.133,.296]	.158	[.097,.218]	.025	.329**	.024
	SRBQ	.103	.014	.283**	[.195,.364]	.213	[.150,.274]	.046		
	Activity Engagement	−.117	.024	−.148**	[−.210, −.081]	−.135	[−.191, −.075]	.018		
	Willingness	−.114	.027	−.144**	[−.222, −.066]	−.116	[−.181, −.055]	.014		
AIS-N	Step 1									
	DBAS	.027	.005	.228**	[.148,.300]				.236**	
	SRBQ	.073	.009	.309**	[.223,.387]					
	Step 2									
	DBAS	.020	.005	.172**	[.085,.254]	.125	[.062,.187]	.016	.250**	.015
	SRBQ	.063	.009	.269**	[.179,.351]	.203	[.137,.264]	.041		
	Activity Engagement	−.046	.017	−.090*	[−.161, −.019]	−.083	[−.147, −.019]	.007		
	Willingness	−.071	.019	−.137**	[−.219, −.052]	−.111	[−.177, −.043]	.012		
AIS-D	Step 1									
	DBAS	.024	.003	.286**	[.204,.363]				.268**	
	SRBQ	.048	.006	.288**	[.204,.374]					
	Step 2									
	DBAS	.019	.003	.230**	[.146,.309]	.167	[.108,.227]	.028	.301**	.033
	SRBQ	.039	.006	.235**	[.149,.322]	.177	[.113,.242]	.031		
	Activity Engagement	−.071	.011	−.194**	[−.255, −130]	−.178	[−.234, −.120]	.032		
	Willingness	−.043	.013	−.119**	[−.202, −.045]	−.096	[−.164, −037]	.009		

*Note*. AIS, Athens Insomnia Scale; AIS-N, Athens Insomnia Scale-Nocturnal; AIS-D, Athens Insomnia Scale-Daytime; DBAS, Dysfunctional Beliefs and Attitudes about Sleep-16; SRBQ, Sleep-Related Behaviors Questionnaire.

*p < 0.01, **p < 0.001.

Next, we categorized the AIS into nocturnal and daytime symptoms and conducted a similar analysis. The Step 1 model for nocturnal symptoms (AIS-N) was statistically significant (*R^2^* = 0.236, *p* < 0.001), indicating a positive association with both DBAS (*β* = 0.228 [0.148, 0.300], *p* < 0.001) and SRBQ (*β* = 0.309 [0.223, 0.387], *p* < 0.001). Step 2 showed a significant increase in explanatory power (*R^2^* = 0.250, *ΔR*^2^ = 0.015, *p* < 0.001). DBAS (*β* = 0.172 [0.085, 0.254], *p* < 0.001, *sr* = 0.125 [0.062, 0.187], *sr^2^* = 0.016), SRBQ (*β* = 0.269 [0.179, 0.351], *p* < 0.001, *sr* = 0.203 [0.137, 0.264], *sr^2^* = 0.041), Activity Engagement (*β* = −0.090 [−0.161, −0.019], *p* = 0.005, *sr* = −0.083 [−0.147, −0.019], *sr²* = 0.007), and Willingness (*β* = −0.137 [−0.219, −0.052], *p* < 0.001, *sr* = −0.111 [−0.177, −0.043], *sr²* = 0.012) were significantly associated with AIS-N.

For daytime symptoms (AIS-D), the Step 1 model was also significant, with positive associations for DBAS (*β* = 0.286 [0.204, 0.363], *p* < 0.001) and SRBQ (*β* = 0.288 [0.204, 0.374], *p* < 0.001) (*R^2^* = 0.268, *p* < 0.001). Step 2 demonstrated a significant increase in explanatory power (*R^2^* = 0.301, *ΔR*^2^ = 0.033, *p* < 0.001). DBAS (*β* = 0.230 [0.146, 0.309], *p* < 0.001, *sr* = 0.167 [0.108, 0.227], *sr^2^* = 0.028), SRBQ (*β* = 0.235 [0.149, 0.322], *p* < 0.001, *sr* = 0.177 [0.113, 0.242], *sr^2^* = 0.031), Activity Engagement (*β* = −0.194 [−0.255, −0.130], *p* < 0.001, *sr* = −0.178 [−0.234, −0.120], *sr²* = 0.032), and Willingness (*β* = −0.119 [−0.202, −0.045], *p* < 0.001, *sr* = −0.096 [−0.164, −0.037], *sr²* = 0.009) were significantly associated with AIS-D.

### Measurement invariance

To assess metric, scalar, and strict invariance across sexes, we conducted a multi-population simultaneous analysis. The configural invariance model for the two-factor model fit well (CFI = 0.971, RMSEA = 0.082 [0.068, 0.096], SRMR = 0.060). The metric invariance model with constraints on factor loadings (ΔCFI = 0.001, ΔRMSEA = −0.007, ΔSRMR = 0.001), the scalar invariance model with additional constraints on item intercepts (ΔCFI = −0.001, ΔRMSEA = −0.004, ΔSRMR = 0.001), and the strict invariance model with additional constraints on residual variances (ΔCFI = −0.002, ΔRMSEA = −0.003, ΔSRMR = 0.000) did not exceed reference values.

To examine age-related metric, scalar, and strict invariance, we conducted another simultaneous multi-population analysis. The configural invariance model also showed good fit (CFI = 0.966, RMSEA = 0.089 [0.072, 0.106], SRMR = 0.064). The metric invariance model with constraints on factor loadings (ΔCFI = 0.000, ΔRMSEA = −0.008, ΔSRMR = 0.004), the scalar invariance model with additional constraints on item intercepts (ΔCFI = −0.002, ΔRMSEA = −0.004, ΔSRMR = 0.002), and the strict invariance model with additional constraints on residual variances (ΔCFI = −0.001, ΔRMSEA = −0.006, ΔSRMR = 0.000) did not exceed reference values.

## Discussion

This study aimed to develop a Japanese version of the SPAQ and assess its reliability and validity in adults with insomnia. These findings indicate that the Japanese SPAQ has good reliability and validity.

### Structural validity

The CFA based on the two-factor model of Bothelius et al. [21] showed a sufficient model fit, with factor loadings exceeding the benchmark values for all items supporting the two-factor structure of the Japanese version.

The CFAs of the one-factor and bifactor models indicated that the one-factor model did not fit well. Although the bifactor model fit indices were acceptable, the factor loadings were positive for Activity Engagement items and negative for willingness items, suggesting that the model may not have adequately captured the common latent construct. This pattern was unexpected because the original SPAQ conceptualized both Activity Engagement and Willingness as reflecting psychological flexibility in insomnia [21]. If both subscales reflect a common aspect of psychological flexibility, some shared variance between them would be expected. However, this was not supported in the present study. Interestingly, the inter-factor correlation between Activity Engagement and Willingness in the Japanese version (*r* = −0.17) was similar to that observed in the original (*r* = −0.26) [21] and Brazilian versions (*r* = −0.10) [28], suggesting that this pattern is not unique to the Japanese version. Therefore, Activity Engagement and Willingness may be better understood as related but distinct constructs rather than as indicators of a single overarching construct. Thus, the structural validity of using the total SPAQ score was not supported. Interpretation should focus on the subscale scores for Activity Engagement and Willingness.

### Reliability

#### Internal consistency.

Cronbach’s α and McDonald’s ω calculations showed that both subscales met the criteria (α ≥ 0.700, ω ≥ 0.700 [32]). The original study [21] also found sufficient values (Activity Engagement: α = 0.89; Willingness: α = 0.73), and our results aligned with these findings. Thus, both subscales of the Japanese SPAQ displayed adequate internal consistency.

#### Test-retest reliability.

The ICCs for test-retest reliability were below the recommended criterion. Therefore, the hypothesis regarding temporal stability was not supported. Because neither the original [21] nor Brazilian versions [28] examined test-retest reliability, this study is the first to evaluate the temporal stability of the SPAQ. Consequently, it remains unclear whether the lower-than-expected ICCs are specific to the Japanese version.

The lower-than-expected ICCs may be explained by fluctuations in Activity Engagement and Willingness, which are influenced by daily life and sleep patterns. Activity Engagement assesses whether individuals can maintain normal daily functioning despite sleep issues, with items such as “I have sleep problems, but my life is going well.” This requires the simultaneous evaluation of self-awareness regarding sleep issues and current daily functioning, which can be affected by changes in sleep patterns and living conditions. Willingness measures the acceptance of sleep problems rather than attempting to control them, exemplified by statements like “controlling sleep problems is the top priority.” This acceptance is heavily influenced by recent sleep quality and insomnia severity. A good night’s sleep may lead to the perception that an item is irrelevant. Therefore, longitudinal variations in SPAQ scores should be interpreted cautiously.

Furthermore, no screening for insomnia diagnostic criteria or treatment status was conducted at Time 2. Therefore, some participants may no longer have met the diagnostic criteria for insomnia or may have received treatment between Time 1 and Time 2. Although the distribution of insomnia severity was similar at the group level (Table 1), changes in insomnia severity and diagnostic status were not examined at the individual level. Such individual variability may have contributed to the lower ICCs. These findings raise the possibility that SPAQ scores may be influenced by changes in sleep status or treatment. Future longitudinal studies that reassess insomnia diagnostic and treatment status at follow-up are needed to clarify the temporal stability of the SPAQ and whether it reflects stable psychological characteristics or state-dependent changes.

### Construct validity

Activity Engagement met the criteria concerning avoidance of experiences, daytime functional impairment, and catastrophic thinking related to insomnia symptoms, whereas willingness met the criteria across all scales. Thus, convergent validity was only partially supported as Activity Engagement satisfied some construct components, whereas willingness met all criteria.

One factor contributing to the smaller correlation observed for Activity Engagement compared to the original study may be differences in sample characteristics. Although participants were recruited from an online panel, the mean AIS score in the present sample (11.96 ± 4.33) was comparable to that of outpatients with insomnia disorder included in the Japanese validation study of the AIS (11.81 ± 4.50) [39]. However, because individuals receiving treatment for physical or mental disorders were excluded from the present study, the sample may have included fewer participants with comorbid conditions and more participants with relatively preserved daily functioning than general clinical populations, despite a comparable level of insomnia severity. Consequently, Activity Engagement, which assesses the ability to continue usual activities despite sleep problems, may have shown weaker associations with insomnia-related measures. Additionally, the strength of the associations involving Activity Engagement differed across the original [21], Brazilian [28], and Japanese versions. For example, the association between Activity Engagement and insomnia severity was weaker in both the Brazilian version (−0.19) and the Japanese version (−0.21) than in the original version (−0.45). These findings suggest that the associations involving Activity Engagement may vary according to sample characteristics or cultural context. Future international comparative studies using samples with comparable characteristics are needed to clarify whether the weaker associations involving Activity Engagement reflect cultural context rather than sample differences.

Previous research [21] found that the effects of the DBAS and SRBQ in Step 1 became insignificant when the SPAQ total score was introduced in Step 2, indicating that only the SPAQ total score significantly explained insomnia symptoms. In contrast, this study found that the explanatory power of the DBAS and SRBQ in Step 2 remained significant. This may be due to stronger associations between the DBAS, SRBQ, and insomnia symptoms in this sample than in the previous study. In the original study, standardized regression coefficients for DBAS and SRBQ in Step 1 were *β* = 0.22 and *β* = 0.23, respectively [21]. In the present study, the coefficients were *β* = 0.279 and *β* = 0.333. Cross-cultural studies have suggested that the association between psychological flexibility and mental health outcomes is weaker in East Asian populations than in Western populations [61,62]. This cultural difference may also have contributed to the discrepancy between the findings of the present study and those of the original study. Although the SPAQ subscales demonstrated statistically significant incremental validity, the increase in explained variance was modest. Future studies should examine the clinical significance of sleep-related psychological flexibility using research designs better suited to evaluating psychological processes, such as mediation analyses and intervention studies.

In analyses that distinguished between nocturnal symptoms and daytime dysfunction, incorporating the SPAQ subscales significantly enhanced the explanatory power. Although Willingness showed minimal differences between nocturnal (*sr*^*2*^ = 0.012) and daytime (*sr*^*2*^ = 0.009) symptoms, Activity Engagement demonstrated greater explanatory power during the day (Nocturnal: *sr*^*2*^ = 0.007, Daytime: *sr*^*2*^ = 0.032). This disparity reflects distinct psychological processes measured by the two subscales. Carmo et al. [28] noted that willingness represents the acceptance of insomnia, correlating consistently with symptoms, regardless of timing. Okajima [29] found that the AAQ-II contributed to both nocturnal (B = −0.07) and daytime (B = −0.12) symptoms in patients with insomnia. Conversely, Activity Engagement signifies a commitment to value-based actions and is related to maintaining daily routines despite sleep issues, linking it to daytime functional impairments. Rash et al. [63] suggested that commitment reduces excessive focus on daytime dysfunction by encouraging value-aligned behavior, which is consistent with the findings of this study. Consequently, the SPAQ may serve as a process indicator within the ACT framework, reflecting both night-time sleep experiences and daytime functioning.

### Measurement invariance

To assess the applicability of the SPAQ across sex and age, a two-factor model enabled simultaneous multi-population analysis. The analysis by sex revealed no significant changes in the fit indices for configural, metric, scalar, and strict invariance. Similarly, the age analysis showed no significant fit changes in the two-factor model. Thus, the Japanese version of the SPAQ is deemed a valid measurement tool across sex and age groups, confirming its measurement invariance.

### Future directions

Activity Engagement and Willingness subscales for SPAQ are negatively correlated, as observed in both the original [21] and Brazilian versions [28]. Both factors were negatively correlated with insomnia-related measures and independently influenced insomnia symptoms. Individuals with insomnia may prioritize either Activity Engagement or Willingness, suggesting that when one is high, the other tends to be low.

A pattern of low psychological flexibility may occur among individuals with high Activity Engagement and low willingness. These individuals believe that they can manage their daily lives despite sleep problems, but that controlling those problems takes precedence. This focus on control hampers the ability to accept unpleasant sleep experiences, indicating high experiential avoidance.

In another pattern, individuals with low Activity Engagement and high willingness accept that sleep problems hinder their well-being but feel no need to control them. This acceptance may restrict engagement in value-aligned activities, leading to avoidance behaviors.

In the context of insomnia, true psychological flexibility involves living with sleep problems and letting go of the need to control them. Consequently, ACT for insomnia should aim to increase Activity Engagement and Willingness.

Future studies should examine whether distinct psychological profiles can be identified based on patterns of Activity Engagement and Willingness scores. This study indicates that, while these concepts are distinct, both must be high for optimal sleep. Low scores indicate impaired psychological flexibility. The ACT offers a flexible framework that is adaptable to various groups [64]. Treatment effectiveness may be improved by tailoring intervention sequences and emphasizing specific processes based on scoring patterns. For example, individuals functioning daily but needing control over their sleep (high Activity Engagement and low willingness) could benefit from a focus on acceptance and defusion, fostering their willingness to let go of control and pursue authentic values. However, research on the SPAQ subscales remains nascent. Future studies should investigate categorization based on lower-order scale score patterns and clarify the clinical characteristics and intervention implications of any identified category.

### Limitations

This study had some limitations. First, it did not assess predictive validity, measurement errors or responsiveness, which are important for establishing the clinical utility of the Japanese version of the SPAQ. The lower-than-expected test-retest reliability indicates that SPAQ scores may be subject to intra-individual variation, highlighting the need to define clinically meaningful changes resulting from interventions. Future longitudinal studies should also examine whether SPAQ-J scores predict subsequent insomnia severity and treatment outcomes. Second, the study relied solely on questionnaire-based measures and did not explore correlations with sleep indicators such as sleep latency, wake after sleep onset, and total sleep time, which were assessed through sleep diaries in the original study [21]. Future studies should also examine the relationships between the SPAQ-J and behavioral sleep variables to provide a more comprehensive understanding of the association between sleep-related psychological flexibility and sleep problems. Third, the sample characteristics and recruitment methods differed from those of the original study [21], which focused on applicants for an insomnia improvement program. This study targeted individuals meeting the insomnia diagnostic criteria but not currently undergoing specialized treatment. Additionally, participants were recruited through an online survey panel, which may have introduced selection bias by overrepresenting individuals with greater interest in sleep problems or greater familiarity with online surveys. Therefore, the generalizability of the present findings to clinical populations is limited. Future studies should further examine the psychometric properties of the SPAQ-J in clinically diagnosed insomnia samples, including individuals with comorbid insomnia and those receiving specialized treatment. Fourth, this study included only individuals who met the insomnia diagnostic criteria based on self-report. In contrast, the Brazilian version of the SPAQ allows participants to report any sleep problems, thereby expanding its applicability [28]. Adopting procedures similar to those of the Brazilian version could broaden the scope of the Japanese version. Fifth, only the AAQ-II was used to assess psychological flexibility when examining convergent validity. Future studies should clarify the aspects of Activity Engagement and Willingness by relating them to other psychological flexibility measures such as value and commitment.

## Conclusion

This study demonstrated acceptable structural validity, internal consistency, and construct validity, along with measurement invariance across sex and age, for the two-factor structure of the Japanese version of the SPAQ. These findings suggest that the SPAQ-J can be used as a measure of psychological flexibility in insomnia research, with careful interpretation of each subscale. However, the lower-than-expected test-retest reliability indicates that the temporal stability of the SPAQ-J scores requires further investigation. Therefore, score stability should be interpreted with caution. Future research should further investigate temporal stability, sample differences, and the clinical utility of the subscale scores.

## Supporting information

S1 Fig One factor and bifactor models of the SPAQ using confirmatory factorial analysis. (TIF)
