# Supplementary material for: Reliability and validity of the Japanese version of the Sleep Problem Acceptance Questionnaire
Source: PLoS One. 2026 Oct 16;21(10):e0358990. doi: 10.1371/journal.pone.0358990 (PMC13674769; doi:10.1371/journal.pone.0358990)
Supplement: Supplementary file 1 [file pone.0358990.s002.docx]

# **Dear Academic Editor and Reviewers:**

# **We would like to thank you for the time you spent reviewing this manuscript. We have found your comments very useful; they have helped us to improve the manuscript significantly. Listed below are the comments you provided and explanations regarding how we have addressed them.**

# **Thank you again for your time and consideration.**

# **【Response to the Academic Editor】**

## **Comment 1**

Please ensure that your manuscript meets PLOS ONE's style requirements, including those for file naming.

**Reply**:

We have checked the manuscript and file names to ensure compliance with PLOS ONE style requirements.

## **Comment 2**

In your ethics statement, please ensure you have provided the full name of the ethics committee(s) that approved this study and specified that approval was granted. If more than 5 committees provided approval, it is acceptable to provide a list as a Supporting Information file.

**Reply**:

We have revised the ethics statement in the manuscript to include the full name of the ethics committee that approved this study, Research Ethics Committee, Graduate School of Human Life Science, Tokyo Kasei University. We have also revised the Ethics Statement in the online submission form accordingly.

## **Comment 3**

Your ethics statement should only appear in the Methods section of your manuscript. If your ethics statement is written in any section besides the Methods, please delete it from any other section.

**Reply**:

We have ensured that the ethics statement appears only in the Methods section.

## **Comment 4**

In the online submission form, you indicated that your data will be submitted to a repository upon acceptance. We strongly recommend all authors deposit their data before acceptance, as the process can be lengthy and hold up publication timelines. Please note that, though access restrictions are acceptable now, your entire minimal dataset will need to be made freely accessible if your manuscript is accepted for publication. This policy applies to all data except where public deposition would breach compliance with the protocol approved by your research ethics board. If you are unable to adhere to our open data policy, please kindly revise your statement to explain your reasoning and we will seek the editor's input on an exemption.

**Reply**:

Thank you for your comment. We would like to clarify that the anonymized dataset and analysis code had already been deposited in the Open Science Framework (OSF) at the time of submission. During peer review, they are accessible via an anonymous view-only link, and the repository will be made publicly accessible by removing the view-only restriction upon publication. We have revised the statement in the online submission form accordingly.

## **Comment 5**

Thank you for stating the following in the Competing Interests/Financial Disclosure section:
Isa Okajima reports receiving grants and consultation fees from NEC Solution Innovators Ltd.; grants from Infocom Co.; lecture fees from Otsuka Pharmaceutical Co. Ltd., MSD LLC, Shionogi & Co., Ltd., Daiichi Sankyo Co., Ltd., Taisho Pharmaceutical Co., LTd., and Eisai Co., Ltd.; and consultation fees from Suntory Wellness Ltd. for projects unrelated to the submitted work.
We note that you received funding from a commercial source: [NEC Solution Innovators Ltd.; Infocom Co.; Otsuka Pharmaceutical Co. Ltd., MSD LLC, Shionogi & Co., Ltd., Daiichi Sankyo Co., Ltd., Taisho Pharmaceutical Co., LTd., and Eisai Co., Ltd.; and Suntory Wellness Ltd.

Please provide an amended Competing Interests Statement that explicitly states this commercial funder, along with any other relevant declarations relating to employment, consultancy, patents, products in development, marketed products, etc.
Within this Competing Interests Statement, please confirm that this does not alter your adherence to all PLOS ONE policies on sharing data and materials by including the following statement: "This does not alter our adherence to PLOS ONE policies on sharing data and materials.” (as detailed online in our guide for authors http://journals.plos.org/plosone/s/competing-interests). If there are restrictions on sharing of data and/or materials, please state these. Please note that we cannot proceed with consideration of your article until this information has been declared.
Please include your amended Competing Interests Statement within your cover letter. We will change the online submission form on your behalf.

**Reply**:

We have revised the Competing Interests Statement and included it in the cover letter, as requested. The revised statement explicitly describes the relevant commercial relationships and includes the required statement confirming that these competing interests do not alter our adherence to PLOS ONE policies on sharing data and materials.

## **Comment 6**

Thank you for providing your underlying data as Supporting Information.
We note that the data set contains text or data that is not in English. Please note that PLOS is an English-language publisher, so we require data sets to be provided in English as well. Please upload an English-language version of your data set.

**Reply**:

Thank you for your comment. We would like to clarify that the file containing Japanese text is not the underlying dataset, but the Japanese version of the Sleep Problem Acceptance Questionnaire (SPAQ-J). The anonymized dataset and analysis code are deposited in the Open Science Framework (OSF), as described in the Data Availability Statement, and were not submitted as Supporting Information. To avoid confusion and comply with the journal's requirements, we have removed the SPAQ-J questionnaire from the Supporting Information and retained it only in the OSF repository.

# **【Response to Reviewer #1】**

## **Comment 1**

The test-retest reliability for both subscales (Activity Engagement: ICC = 0.586; Willingness: ICC = 0.589) fell substantially below the accepted threshold of 0.700. While the authors provide a reasonable explanation that these constructs may fluctuate with daily sleep patterns and insomnia severity, this finding raises fundamental questions about the stability of the measure. The authors should consider whether the SPAQ-J is truly measuring a trait-like construct (psychological flexibility) or a state-like construct that varies considerably over time. If the latter, the instrument may be more appropriate for measuring treatment response than for stable individual differences. The authors should discuss whether the original SPAQ has demonstrated adequate test-retest reliability in other populations or whether this is a unique finding of the Japanese version. Additionally, the authors should report whether participants' insomnia status or treatment received between Time 1 and Time 2 was assessed, as this could explain the low ICCs.

**Reply**:

Thank you for the insightful comment. We agree that the lower-than-expected test–retest reliability warrants further discussion. We have substantially revised the "Test–Retest Reliability" section of the Discussion. First, we clarified that neither the original SPAQ nor the Brazilian version examined test–retest reliability. Therefore, this study is the first to evaluate the temporal stability of the SPAQ, and it remains unclear whether the lower ICCs are specific to the Japanese version.

Second, we explicitly clarified that screening for insomnia diagnostic criteria and treatment status was not conducted at Time 2, although insomnia severity (AIS) was reassessed. We also noted that the distribution of insomnia severity was similar at the group level across the two assessment points. However, because individual-level changes in insomnia severity were not examined and information on diagnostic status and treatment was unavailable at Time 2, we discussed the possibility that some participants may no longer have met the diagnostic criteria for insomnia or may have received treatment between assessments, which could have contributed to the lower ICCs.

Finally, we expanded the discussion regarding the interpretation of the lower ICCs. We acknowledged the possibility that the SPAQ may be sensitive to state-dependent changes in sleep status rather than solely reflecting stable individual differences. However, because the present study was not designed to distinguish trait-like from state-like characteristics, we refrained from drawing a definitive conclusion and instead highlighted the need for future longitudinal and intervention studies to clarify whether the SPAQ primarily reflects stable psychological flexibility or state-dependent changes.

**Revised**:

**L480~484:** Therefore, the hypothesis regarding temporal stability was not supported. Because neither the original [21] nor Brazilian versions [28] examined test-retest reliability, this study is the first to evaluate the temporal stability of the SPAQ. Consequently, it remains unclear whether the lower-than-expected ICCs are specific to the Japanese version.
**L495~504:** Furthermore, no screening for insomnia diagnostic criteria or treatment status was conducted at Time 2. Therefore, some participants may no longer have met the diagnostic criteria for insomnia or may have received treatment between Time 1 and Time 2. Although the distribution of insomnia severity was similar at the group level (Table 1), changes in insomnia severity and diagnostic status were not examined at the individual level. Such individual variability may have contributed to the lower ICCs. These findings raise the possibility that SPAQ scores may be influenced by changes in sleep status or treatment. Future longitudinal studies that reassess insomnia diagnostic and treatment status at follow-up are needed to clarify the temporal stability of the SPAQ and whether it reflects stable psychological characteristics or state-dependent changes.

## **Comment 2**

The authors report that the bifactor model showed adequate fit indices (CFI = 0.998, RMSEA = 0.024, SRMR = 0.012) but conclude that the total score is not supported because the general factor loadings were positive for Activity Engagement items and negative for Willingness items. While this pattern indeed suggests that a single common factor is not appropriate, the authors should provide more detailed explanation of this decision. The negative loadings in a bifactor model can indicate that the two subscales represent opposing constructs rather than a unified construct. The authors should consider whether a hierarchical model might be more appropriate or whether the two subscales should simply be treated as separate but related constructs. Furthermore, the authors should clarify whether the negative correlation between subscales (r = -0.17) is consistent with the original version and whether this pattern was expected given the theoretical framework of the SPAQ.

**Reply**:

Thank you for your meaningful comment regarding the interpretation of the bifactor model. We agree that a more detailed explanation of this decision is warranted, and we have revised the "Structural Validity" section of the Discussion accordingly.

We did examine a higher-order factor model, as suggested. However, because the correlation between the two first-order factors was negative, the higher-order model produced an improper solution—specifically, a negative residual variance for the general factor and a markedly inflated standard error for the Willingness loading—indicating that a higher-order factor could not be meaningfully estimated from two negatively correlated first-order factors. Therefore, we concluded that the higher-order model is not statistically supportable in this dataset, and we have not reported these results in the manuscript, as they are redundant.

Additionally, we clarified that the negative correlation between Activity Engagement and Willingness was consistent with both the original SPAQ (*r* = - 0.26) and the Brazilian version (*r* = - 0.10). We further discussed that this pattern was not consistent with the theoretical assumption that both subscales reflect a common aspect of psychological flexibility. Therefore, we suggested that Activity Engagement and Willingness may be better interpreted as related but distinct constructs rather than indicators of a single overarching construct, and that interpretation should focus on the two subscale scores rather than the total SPAQ score.

**Revised**:

**L460~469:** This pattern was unexpected because the original SPAQ conceptualized both Activity Engagement and Willingness as reflecting psychological flexibility in insomnia [21]. If both subscales reflect a common aspect of psychological flexibility, some shared variance between them would be expected. However, this was not supported in the present study. Interestingly, the inter-factor correlation between Activity Engagement and Willingness in the Japanese version (r = −0.17) was similar to that observed in the original (r = −0.26) [21] and Brazilian versions (r = −0.10) [28], suggesting that this pattern is not unique to the Japanese version. Therefore, Activity Engagement and Willingness may be better understood as related but distinct constructs rather than as indicators of a single overarching construct.

## **Comment 3**

The authors recruited participants with insomnia disorder based on self-reported symptoms meeting DSM-5 criteria, but the sample was drawn from an online panel rather than a clinical setting. The authors acknowledge this limitation but do not fully address its implications. The study excluded individuals receiving treatment for physical or mental disorders, which may have resulted in a sample with less severe insomnia or different psychological characteristics compared to clinical populations. The authors should discuss how the sample characteristics (e.g., 46.8% male, mean age 45.4 years) compare to epidemiological data on insomnia and whether the findings are generalizable to clinical populations seeking treatment. Additionally, the authors should consider whether the weaker correlations observed for Activity Engagement compared to the original study might be attributable to this sample difference and discuss this more thoroughly.

**Reply**:

Thank you for the valuable comment. We agree that a more thorough discussion of the sample characteristics and their implications for generalizability was needed, and we have revised the manuscript accordingly.

First, we added severity-stratified information to Table 1 (Sociodemographic data of participants) based on AIS severity categories, which shows that more than half of the sample had moderate or greater insomnia severity, clarifying that the sample was not predominantly composed of mild cases.

Second, we expanded the "Construct Validity" section of the Discussion to address the weaker correlation observed for Activity Engagement compared with the original study. We noted that, although participants were recruited from an online panel, the mean AIS score in the present sample (11.96 ± 4.33) was comparable to that of outpatients with insomnia disorder in the Japanese validation study of the AIS (11.81 ± 4.50), suggesting that the insomnia severity of the present sample was not lower than that of a general clinical population. However, we also noted that because individuals receiving treatment for physical or mental disorders were excluded from the present study, the sample may have included fewer participants with comorbid conditions and more participants with relatively preserved daily functioning than general clinical populations, despite comparable insomnia severity. We suggested that this difference in sample composition, rather than insomnia severity per se, may partly explain the weaker Activity Engagement correlations relative to the original study.

Through these revisions, we have clarified that while the present sample was comparable to clinical populations in terms of insomnia severity, the exclusion of individuals with comorbid physical or mental disorders may limit the generalizability of the findings to clinical populations with such comorbidities.

**Revised**:

**L512~521:** Although participants were recruited from an online panel, the mean AIS score in the present sample (11.96 ± 4.33) was comparable to that of outpatients with insomnia disorder included in the Japanese validation study of the AIS (11.81 ± 4.50) [39]. However, because individuals receiving treatment for physical or mental disorders were excluded from the present study, the sample may have included fewer participants with comorbid conditions and more participants with relatively preserved daily functioning than general clinical populations, despite a comparable level of insomnia severity. Consequently, Activity Engagement, which assesses the ability to continue usual activities despite sleep problems, may have shown weaker associations with insomnia-related measures.

## **Comment 4**

The authors use a criterion of r ≥ 0.300 based on 95% confidence intervals to determine "moderate or stronger" associations for convergent validity. However, many of the correlations for Activity Engagement fell below this threshold (e.g., with AIS total: r = -0.210; DBAS: r = -0.163; SRBQ: r = -0.199). The authors conclude that convergent validity was "only partially supported" for Activity Engagement. While this is an honest assessment, the authors should more critically discuss whether this indicates that Activity Engagement is less relevant to insomnia-related constructs than Willingness, or whether it reflects limitations of the Activity Engagement subscale itself. The authors should also consider whether the direction of correlations (all negative) is appropriate given that higher SPAQ scores indicate greater psychological flexibility, which should be inversely related to measures of dysfunction. The lack of positive correlations with any measure is notable and should be discussed.

**Reply**:

We agree that the correlations for Activity Engagement falling below the threshold warrant more careful consideration, and we have revised the "Construct Validity" section of the Discussion accordingly.

As noted in our response to Comment 3, the exclusion of individuals receiving treatment for physical or mental disorders in the present study may have resulted in fewer participants with comorbid conditions. We discussed that this may have led to a sample with relatively preserved daily functioning, which could be one reason for the weaker association between Activity Engagement and insomnia-related measures.

We also discussed a limitation of the Activity Engagement subscale itself, noting that the strength of associations involving Activity Engagement differed across the original, Brazilian, and Japanese versions, and that these associations may be influenced by cultural context, warranting further research.

Regarding the direction of the correlations, all other measures used for convergent validity are scored such that higher scores indicate greater difficulties, whereas higher SPAQ scores reflect greater psychological flexibility. Negative correlations between the SPAQ and these measures were therefore the expected direction, consistent with the scoring of both instruments, and we have clarified this point in the "Construct Validity Verification" section of the Hypotheses.

**Revised**:

**L521~528:** Additionally, the strength of the associations involving Activity Engagement differed across the original [21], Brazilian [28], and Japanese versions. For example, the association between Activity Engagement and insomnia severity was weaker in both the Brazilian version (- 0.19) and the Japanese version (- 0.21) than in the original version (- 0.45). These findings suggest that the associations involving Activity Engagement may vary according to sample characteristics or cultural context. Future international comparative studies using samples with comparable characteristics are needed to clarify whether the weaker associations involving Activity Engagement reflect cultural context rather than sample differences.

## **Comment 5**

The hierarchical regression analyses showed that the SPAQ subscales added only modest incremental explanatory power (ΔR² = 0.024 for AIS total, 0.015 for AIS-N, and 0.033 for AIS-D). While these changes were statistically significant, the practical significance is questionable. The semi-partial correlation squared (s*r*²) values for both SPAQ subscales were small (0.018 and 0.014 for total AIS), indicating that each subscale explained less than 2% of unique variance in insomnia symptoms. The authors should discuss whether these small effect sizes are clinically meaningful and whether the SPAQ provides sufficient additional information beyond existing measures (DBAS and SRBQ) to justify its use in clinical practice. The authors might consider comparing the incremental validity of the SPAQ to the AAQ-II to determine whether sleep-specific acceptance provides advantages over general psychological flexibility.

**Reply**:

Thank you for your useful comment. As already noted in the manuscript, the original study found that the effects of DBAS and SRBQ in Step 1 became nonsignificant once the SPAQ total score was entered in Step 2, whereas in the present study, DBAS and SRBQ remained significant in Step 2. We attributed this to stronger associations among DBAS, SRBQ, and insomnia symptoms in the present sample than in the original study. To further support this interpretation, we added a discussion citing cross-cultural literature suggesting that associations between psychological flexibility and mental health outcomes tend to be weaker in East Asian populations than in Western populations, and we discussed the possibility that this cultural difference may also have contributed to the discrepancy between the present findings and those of the original study.

We would like to note that, even against this background of relatively strong explanatory power of DBAS and SRBQ, the SPAQ subscales still demonstrated statistically significant incremental validity, explaining unique variance in insomnia symptoms beyond these existing measures; we consider this finding itself to be meaningful. At the same time, we agree that the increase in explained variance was modest. We have added a statement that future studies should examine the clinical significance of sleep-related psychological flexibility using research designs better suited to evaluating psychological processes, such as mediation analyses and intervention studies.

Furthermore, we conducted additional hierarchical regression analyses examining the incremental validity of the SPAQ beyond the AAQ-II. As this specific comparison was not part of the original validation study or our planned analyses, we did not incorporate it into the revised manuscript. However, we report the results here for the reviewer's reference.

Three models were examined (Tables A-C below). In Model A, DBAS and SRBQ were entered in Step 1, AAQ-II in Step 2, and the SPAQ subscales in Step 3. The AAQ-II did not significantly predict insomnia symptoms after controlling for DBAS and SRBQ, whereas entering the SPAQ subscales in Step 3 produced a further significant increase in explained variance (*ΔR*² = .021), with both Activity Engagement and Willingness remaining significant predictors alongside DBAS and SRBQ. To directly compare the incremental validity of the AAQ-II and the SPAQ, Model B entered the AAQ-II in Step 1 followed by the SPAQ subscales in Step 2, and Model C reversed this order. In Model B, adding the SPAQ subscales after the AAQ-II produced a significant increase in explained variance (*ΔR*² = .094). In Model C, adding the AAQ-II after the SPAQ subscales also produced a significant increase (*ΔR*² = .035), though smaller in magnitude. Thus, while both measures explained some unique variance beyond the other, the SPAQ subscales accounted for substantially more additional variance in insomnia symptoms than the AAQ-II did. These results suggest that the SPAQ captures aspects of sleep-related psychological flexibility not fully accounted for by a measure of general psychological flexibility.

**Revised**:

**L536~543:** Cross-cultural studies have suggested that the association between psychological flexibility and mental health outcomes is weaker in East Asian populations than in Western populations [61, 62]. This cultural difference may also have contributed to the discrepancy between the findings of the present study and those of the original study. Although the SPAQ subscales demonstrated statistically significant incremental validity, the increase in explained variance was modest. Future studies should examine the clinical significance of sleep-related psychological flexibility using research designs better suited to evaluating psychological processes, such as mediation analyses and intervention studies.

## **Comment 6**

Add "This limitation has prompted interest in non-pharmacological interventions aimed at improving sleep, which encompass a variety of strategies, including sleep hygiene." after CBT-I weakness.

References: Alghosi M, Sharifi M, Namavari S, Rajamand N, Bamorovat F, Norouzi N, Alimoradi M and Konrad A (2025) The effect of chronic yoga interventions on sleep quality in people with sleep disorders: a scoping review. Front. Neurol. 16:1566445. doi: 10.3389/fneur.2025.1566445

Mohammad, A., Elham, H. & Andreas, K. A scoping review of the effect of chronic stretch training on sleep quality in people with sleep disorders. Eur J Appl Physiol 124, 2533–2545 (2024). https://doi.org/10.1007/s00421-024-05541-z

**Reply**:

**Thank you** for your valuable suggestion. We agree that expanding on this point strengthens the Introduction. However, we would like to note that a recent component network meta-analysis (Furukawa et al., 2024, JAMA Psychiatry) found that sleep hygiene education, a component often used to represent non-pharmacological interventions, was not associated with improved sleep quality, while relaxation techniques were potentially detrimental. Given this evidence, we were hesitant to specifically highlight sleep hygiene as a representative example of promising non-pharmacological approaches, as this could be interpreted as inconsistent with the current evidence base.

Instead, to address the reviewer's broader point regarding the limitations of CBT-I and the resulting interest in complementary approaches, we revised the manuscript to incorporate one of the suggested references (Alghosi et al., 2025), which reports that yoga alleviates daytime dysfunction in individuals with sleep disorders, while focusing on the daytime symptom limitation specifically discussed in this paragraph. We believe this revision addresses the reviewer's suggestion regarding the introduction of relevant literature while maintaining consistency with the current evidence base.

**Revised**:

**L45~48:** This limitation has led to the proposal of various interventions aiming to comprehensively address daytime symptoms as well [9]. Among these, Acceptance and Commitment Therapy (ACT [10]) has attracted growing attention in recent years.

# **【Response to Reviewer #2】**

The manuscript reports ICC values of approximately 0.59 for both subscales, which are below the commonly accepted threshold of 0.70. Although this limitation is acknowledged, the Discussion tends to minimize its importance. The authors should provide a more detailed explanation for the relatively low temporal stability and discuss possible reasons, including characteristics of the construct, sample variability, and the two-week interval.

**Reply**:

Thank you for the useful comment. As also addressed in our response to Reviewer 1's Comment 1, we agree that the Discussion should provide a more detailed explanation for the low ICCs, and we have substantially revised the "Test-Retest Reliability" section accordingly.

Regarding the characteristics of the construct, the original manuscript already discussed that Activity Engagement and Willingness may be inherently susceptible to short-term fluctuation, as they require participants to evaluate their current daily functioning and acceptance of sleep problems, both of which can shift with recent sleep patterns and living conditions. Based on this, we noted that longitudinal variations in SPAQ scores should be interpreted with caution. However, we agree that this alone was insufficient as a discussion of test-retest reliability, and we therefore added the following.

Regarding sample variability, we described that diagnostic status and treatment status were not reassessed at Time 2, although insomnia severity (AIS) was done. While the distribution of insomnia severity was similar at the group level, individual-level changes in severity or diagnostic status were not examined. We discussed that during the two-week interval between Time 1 and Time 2, some participants may no longer have met the diagnostic criteria for insomnia or may have received treatment, which could have contributed to the lower ICCs.

Additionally, we described that because neither the original nor the Brazilian version examined test–retest reliability, this is the first study to evaluate the temporal stability of the SPAQ, and it remains unclear whether the lower ICCs are unique to the Japanese version. We have added a recommendation that future longitudinal and intervention studies are needed to further clarify whether the SPAQ primarily reflects stable psychological characteristics or state-dependent changes.

**Revised**:

**L480~484:** Therefore, the hypothesis regarding temporal stability was not supported. Because neither the original [21] nor Brazilian versions [28] examined test-retest reliability, this study is the first to evaluate the temporal stability of the SPAQ. Consequently, it remains unclear whether the lower-than-expected ICCs are specific to the Japanese version.
**L495~504:** Furthermore, no screening for insomnia diagnostic criteria or treatment status was conducted at Time 2. Therefore, some participants may no longer have met the diagnostic criteria for insomnia or may have received treatment between Time 1 and Time 2. Although the distribution of insomnia severity was similar at the group level (Table 1), changes in insomnia severity and diagnostic status were not examined at the individual level. Such individual variability may have contributed to the lower ICCs. These findings raise the possibility that SPAQ scores may be influenced by changes in sleep status or treatment. Future longitudinal studies that reassess insomnia diagnostic and treatment status at follow-up are needed to clarify the temporal stability of the SPAQ and whether it reflects stable psychological characteristics or state-dependent changes.

# **【Response to Reviewer #3】**

## **Comment 1: Abstract**

It would benefit from including more quantitative information regarding the psychometric performance of the instrument. Specifically, the authors should report the key confirmatory factor analysis (CFA) fit indices and the actual test–retest reliability coefficient rather than stating only that it was below the recommended threshold. In addition, briefly mentioning the translation and cross-cultural adaptation process would provide a more complete overview of the study.

**Reply**:

Thank you for the meaningful comment. We agree that the Abstract would benefit from more specific quantitative information, and we have revised it accordingly. Specifically, we added the key CFA fit indices, the internal consistency values, and the actual test-retest reliability coefficients, rather than only stating that the latter fell below the recommended threshold.

Regarding the translation and cross-cultural adaptation process, we added a brief description to the opening of the Abstract, noting that the SPAQ-J was developed through a forward–backward translation procedure with permission from the original authors.

**Revised**:

**L16~17:** The SPAQ-J was developed through a forward-backward translation procedure with permission from the original authors.

**L21~25:** the original version (CFI = 0.972, TLI = 0.958, RMSEA = 0.082, SRMR = 0.059): Activity Engagement and Willingness. Although internal consistency was adequate (Activity Engagement: α = 0.868, ω = 0.870; Willingness: α = 0.895, ω = 0.895), test-retest reliability was below the recommended threshold (Activity Engagement: ICC = 0.586, Willingness: ICC = 0.589).

## **Comment 2: Introduction**

The rationale for developing a Japanese version of the SPAQ could be strengthened. The authors should more clearly articulate the specific research gap and explain why validation in the Japanese population is necessary despite the availability of the original and other translated versions. Furthermore, a brief discussion explaining why the SPAQ was selected over other ACT-related measures would further justify the study.

**Reply**:

We agree that the rationale needed to be strengthened, and we have revised the Introduction accordingly. Regarding the choice of the SPAQ, we clarified that it is the only sleep-specific measure of psychological flexibility that has been adopted in multiple ACT intervention studies for insomnia. Regarding the research gap, we clarified that the SPAQ has only been validated in Brazilian Portuguese, with no Japanese version available, and that prior studies in Japan have linked psychological flexibility to insomnia severity, underscoring the need to assess this construct in the Japanese population.

**Revised**:

**L82~91:** Sleep-specific measures of psychological flexibility are currently limited to the SPAQ and the Sleep Acceptance Scale (SAS) [25]. Among these, the SPAQ is the only measure that has been adopted in multiple ACT intervention studies for insomnia [26,27]. However, besides the original version, the SPAQ has only been translated and validated in a Brazilian Portuguese version [28], and no validated Japanese version is available. Previous studies in Japan have reported associations between psychological flexibility and insomnia severity [29], highlighting the importance of assessing this construct in the context of sleep. A validated Japanese version of the SPAQ would facilitate future ACT for insomnia research and clinical evaluation in Japan while enabling findings from Japanese studies to be interpreted alongside the growing international literature.

## **Comment 3: Hypotheses**

The hypotheses are presented within the text but would benefit from being stated more explicitly. Presenting each hypothesis separately (e.g., H1, H2, H3, etc.) would improve clarity and help readers better understand the specific objectives and expected outcomes of the study.

**Reply**:

We agree that presenting the hypotheses more explicitly would improve clarity, and we have revised the Hypotheses section accordingly, stating each hypothesis separately (H1~ H5) alongside its corresponding analysis, organized under the headings "Structural Validity Verification," "Reliability Verification," "Construct Validity Verification," and "Verification of Measurement Invariance."

**Revised**:

**L105:** H1. The SPAQ-J would retain the original two-factor structure of the SPAQ.

**L113:** H2. The SPAQ-J would demonstrate satisfactory internal consistency and test–retest reliability.

**L120~121:** H3. The SPAQ-J would show moderate associations with theoretically related constructs, supporting convergent validity.

**L129~130:** H4. The SPAQ-J factors would explain additional variance in insomnia symptoms beyond dysfunctional sleep beliefs and sleep-related safety behaviors, supporting incremental validity.

**L135:** H5. The SPAQ-J would demonstrate measurement invariance across sex and age groups.

## **Comment 4: Participants**

The exclusive use of an online survey panel may introduce selection bias. The authors should discuss the potential limitations associated with recruiting participants through an online panel and address how representative this sample is of the broader Japanese population with insomnia. Additionally, more information regarding the response rate and participant exclusion process would improve transparency.

**Reply**:

Thank you for the useful comment. As this comment spans several sections, we address each in the order they appear in the manuscript.

Regarding the response rate and exclusion process, this information was previously presented only in Fig 1 (Flowchart of the participant selection for this study). In response to this comment, we have added a description of the participant selection flow in the text, in the "Research Participants and Procedures" section of Methods. Additionally, we added severity-stratified sociodemographic information to Table 1 based on AIS severity categories, showing the distribution of insomnia severity within the present sample. Furthermore, in the "Construct Validity" section of the Discussion, we noted that the mean AIS score of the present sample was comparable to that of outpatients with insomnia disorder in the Japanese validation study of the AIS, suggesting that insomnia severity did not differ substantially from a general clinical population. Additionally, we discussed that the exclusion of individuals already receiving treatment may have resulted in fewer participants with secondary insomnia.

Finally, in the Limitations section, we added a statement acknowledging that recruitment through an online survey panel may have introduced selection bias by overrepresenting individuals with greater interest in sleep problems or greater familiarity with online surveys; therefore, our findings may limit the generalizability of the findings to clinical populations.

**Revised**:

**L155~160:** The survey company distributed the online survey to 409,232 registered panelists. After excluding individuals who did not meet the eligibility criteria based on the screening items or did not complete the survey, 1,422 participants completed the survey. Among them, 561 were excluded due to incomplete or invalid responses, resulting in 861 participants at Time 1. At Time 2 (two weeks later), 490 participants were excluded due to non-response or invalid responses, resulting in 371 participants at Time 2. The participant recruitment process is illustrated in Fig 1.

## **Comment 5: Translation Procedure**

The translation procedure appears rigorous and follows standard forward-backward translation methods. Nevertheless, it would be valuable to clarify whether cognitive debriefing, pilot testing, or interviews with members of the target population were conducted to ensure item clarity, cultural appropriateness, and conceptual equivalence before the final version was established.

**Reply**:

Thank you for your valuable comment. We have revised the Research Materials section to provide additional details regarding the translation procedure. Specifically, we clarified that cognitive debriefing was conducted with several Japanese adults, who completed the translated items and provided feedback on item clarity and the naturalness of the wording. This process confirmed that the final translated items posed no issues in terms of clarity and naturalness of the wording. We also explicitly stated that pilot testing and interviews with individuals with insomnia were not conducted prior to the main survey.

**Revised**:

**L188~192:** Subsequently, cognitive debriefing was conducted with several Japanese adults, who individually completed the translated items and provided verbal feedback on item clarity and naturalness of the wording. This process confirmed that the item content posed no issues. However, pilot testing and interviews with individuals with insomnia were not conducted prior to the main survey. This led to the creation of the final version (Appendix Table 1).

## **Comment 6: Statistical Analysis**

Several methodological details should be clarified. The authors should report whether the assumptions underlying Pearson correlation and multiple regression analyses were evaluated, including normality, linearity, homoscedasticity, and multicollinearity. In addition, more information regarding missing data, including the proportion of missing observations and the rationale for using Full Information Maximum Likelihood (FIML), would improve methodological transparency.

**Reply**:

Thank you for the meaningful comment. We have revised the manuscript to clarify the assumptions underlying the Pearson correlation and multiple regression analyses. Specifically, we added descriptions of the procedures used to evaluate normality, linearity, homoscedasticity, and multicollinearity, together with the corresponding evaluation criteria. We also report the results of these assumption checks, indicating that no substantial violations of normality, linearity, or multicollinearity were identified. Although the Breusch-Pagan test suggested mild heteroscedasticity, sensitivity analyses using HC3 robust standard errors yielded the same substantive conclusions as the primary analyses.

Additionally, we clarified the handling of missing data. We explicitly state that the dataset provided by the survey company contained no missing values because incomplete responses had already been excluded prior to delivery. We also clarified that, although full information maximum likelihood (FIML) was specified for the CFA, it produced identical estimates to standard maximum likelihood estimation because the analyzed dataset contained no missing values.

**Revised**:

**L252~254:** The dataset delivered by the survey company contained no missing values, as responses with incomplete or missing items had already been excluded prior to delivery.

**L256~258:** For sample sizes greater than 300, absolute skewness values below 2 and absolute kurtosis values below 7 were considered indicative of an approximately normal distribution [50].

**L262~265:** Although full information maximum likelihood (FIML) was specified in the analysis code, the dataset contained no missing values; therefore, FIML yielded the same estimates as standard maximum likelihood estimation.

**L284~285:** Linearity was evaluated by visual inspection of scatterplots for each variable pair prior to the correlation analyses.

**L298~304:** Prior to the regression analyses, residual normality was evaluated using Q-Q plots. Linearity and homoscedasticity were assessed by visual inspection of residual-versus-fitted plots, and homoscedasticity was additionally evaluated using the Breusch-Pagan test [58]. Multicollinearity was assessed using variance inflation factors (VIFs), with values below 5 considered acceptable [59]. When the Breusch–Pagan test indicated heteroscedasticity, HC3 robust standard errors were additionally applied as a sensitivity analysis to account for potential heteroscedasticity [60].
**L321:** All variables met the predefined criteria for approximate normality.

**L367~368:** Visual inspection of scatterplots indicated no substantial deviations from linearity for any variable pair.

**L392~397:** Visual inspection of the Q-Q plots indicated no substantial violations of residual normality, whereas inspection of the residual-versus-fitted plots indicated no substantial violations of linearity or homoscedasticity. Although the Breusch–Pagan test indicated mild heteroscedasticity in all three models, sensitivity analyses based on HC3 robust standard errors yielded the same substantive conclusions as the primary analyses. VIFs values ranged from 1.19 to 1.89 across all models, indicating no evidence of multicollinearity.

## **Comment 7: Results**

Given that the test–retest reliability did not reach the recommended threshold, it would be helpful to report confidence intervals alongside all reliability estimates and briefly highlight this limitation within the Results section before discussing it in greater detail later. Reporting potential floor and ceiling effects, if examined, would also strengthen the psychometric evaluation.

**Reply**:

The Results section already stated that “the ICC (2,1) values for Activity Engagement and Willingness were below the reference ranges,” which was intended to indicate that test-retest reliability did not reach the recommended level. We have retained this statement in the revised manuscript. Should further clarification be considered necessary, we would be pleased to revise the wording accordingly.

Additionally, we added 95% confidence intervals for Cronbach’s α and McDonald’s ω and included a new Floor and Ceiling Effects subsection, reporting that no floor or ceiling effects were observed for either subscale.

**Revised**:

**L270~273:** Floor and ceiling effects were examined for the SPAQ item groups by calculating the percentage of participants who obtained the lowest or highest possible score. Floor or ceiling effects were considered present if more than 15% of participants achieved the lowest or highest possible score, respectively [53].
**L352~355:** Floor and ceiling effects were examined for each subscale. No floor or ceiling effects were observed for either the Activity Engagement or Willingness subscale, with 2.7% and 2.2% of participants scoring at the minimum and maximum possible values for Activity Engagement, respectively, and 2.6% and 1.5% for Willingness.
**L359~360:** Activity Engagement: α = 0.868 [0.853, 0.882], ω = 0.870 [0.849, 0.887]; Willingness: α = 0.895 [0.883, 0.906], ω = 0.895 [0.881, 0.910]

## **Comment 8: Discussion**

Several aspects could be expanded. First, the relatively low test–retest reliability deserves a more thorough explanation, including possible methodological or cultural reasons for this finding and its implications for future use of the instrument. Second, the discussion would benefit from a deeper comparison with previous validation studies conducted in other languages, particularly similarities and differences in psychometric performance. Finally, the potential influence of Japanese cultural characteristics on acceptance-related constructs and psychological flexibility could be discussed in greater depth.

**Reply**:

Thank you for the useful comment. We have substantially expanded the Discussion to address these important points. First, we provided a more detailed interpretation of the relatively low test-retest reliability by discussing potential methodological factors that may have contributed to the findings. In particular, we clarified that insomnia diagnostic status and treatment status were not reassessed at Time 2, and that individual changes in these factors may have contributed to the lower-than-expected ICCs. We also clarified that test-retest reliability has not been examined in the original or Brazilian versions of the SPAQ; therefore, it remains unclear whether the lower ICCs are specific to the Japanese version.

Second, we strengthened comparisons with previous language versions of the SPAQ throughout the Discussion by expanding the comparison of the present findings with those of the original and Brazilian versions across multiple psychometric properties, including structural validity, test-retest reliability, and construct validity.

Finally, we expanded the discussion in the Construct Validity section concerning the findings of the hierarchical multiple regression analyses by incorporating evidence from cross-cultural research. Specifically, we discussed evidence suggesting that the association between psychological flexibility and mental health outcomes tends to be weaker in East Asian populations than in Western populations, and considered the possibility that this cultural difference may have contributed to the discrepancy between the findings of the present study and those of the original study.

**Revised**:

**L480~484:** Therefore, the hypothesis regarding temporal stability was not supported. Because neither the original [21] nor Brazilian versions [28] examined test-retest reliability, this study is the first to evaluate the temporal stability of the SPAQ. Consequently, it remains unclear whether the lower-than-expected ICCs are specific to the Japanese version.
L495~504: Furthermore, no screening for insomnia diagnostic criteria or treatment status was conducted at Time 2. Therefore, some participants may no longer have met the diagnostic criteria for insomnia or may have received treatment between Time 1 and Time 2. Although the distribution of insomnia severity was similar at the group level (Table 1), changes in insomnia severity and diagnostic status were not examined at the individual level. Such individual variability may have contributed to the lower ICCs. These findings raise the possibility that SPAQ scores may be influenced by changes in sleep status or treatment. Future longitudinal studies that reassess insomnia diagnostic and treatment status at follow-up are needed to clarify the temporal stability of the SPAQ and whether it reflects stable psychological characteristics or state-dependent changes.

**L521~528:** Additionally, the strength of the associations involving Activity Engagement differed across the original [21], Brazilian [28], and Japanese versions. For example, the association between Activity Engagement and insomnia severity was weaker in both the Brazilian version (- 0.19) and the Japanese version (- 0.21) than in the original version (- 0.45). These findings suggest that the associations involving Activity Engagement may vary according to sample characteristics or cultural context. Future international comparative studies using samples with comparable characteristics are needed to clarify whether the weaker associations involving Activity Engagement reflect cultural context rather than sample differences.

**L536~543:** Cross-cultural studies have suggested that the association between psychological flexibility and mental health outcomes is weaker in East Asian populations than in Western populations [61, 62]. This cultural difference may also have contributed to the discrepancy between the findings of the present study and those of the original study. Although the SPAQ subscales demonstrated statistically significant incremental validity, the increase in explained variance was modest. Future studies should examine the clinical significance of sleep-related psychological flexibility using research designs better suited to evaluating psychological processes, such as mediation analyses and intervention studies.

## **Comment 9: Limitations**

These include the use of self-reported measures, potential selection bias resulting from online recruitment, the absence of clinically diagnosed insomnia cases, and the lack of assessment of predictive validity and responsiveness to treatment. A more comprehensive discussion of these issues would provide a balanced interpretation of the findings.

**Reply**:

We expanded the Limitations section to provide a more comprehensive discussion of the limitations of the present study and directions for future research. Specifically, we acknowledged that predictive validity and responsiveness to treatment were not evaluated and emphasized the need for future longitudinal studies to examine whether SPAQ-J scores predict subsequent insomnia severity, treatment outcomes, and clinically meaningful changes following intervention. We also noted that the study relied exclusively on self-report questionnaires and did not include behavioral sleep variables, such as sleep diary measures, highlighting the need to examine the relationship between the SPAQ-J and objective or behavioral sleep-related variables in future research. In addition, we expanded the discussion of the study sample by acknowledging the potential selection bias associated with recruitment through an online survey panel and the resulting limitations in generalizability. We further emphasized the need for future validation studies in clinically diagnosed insomnia samples, including individuals with comorbid insomnia and those receiving specialized treatment.

**Revised**:

**L599~504:** This study had several limitations. First, it did not assess predictive validity, measurement errors or responsiveness, which are important for establishing the clinical utility of the Japanese version of the SPAQ. The lower-than-expected test-retest reliability indicates that SPAQ scores may be subject to intra-individual variation, highlighting the need to define clinically meaningful changes resulting from interventions. Future longitudinal studies should also examine whether SPAQ-J scores predict subsequent insomnia severity and treatment outcomes.

**L607~610:** Future studies should also examine the relationships between the SPAQ-J and behavioral sleep indicators to provide a more comprehensive understanding of the association between sleep-related psychological flexibility and sleep problems.

**L613~619:** In addition, participants were recruited through an online survey panel, which may have introduced selection bias by overrepresenting individuals with greater interest in sleep problems or greater familiarity with online surveys. Therefore, the generalizability of the present findings to clinical populations is limited. Future studies should further examine the psychometric properties of the SPAQ-J in clinically diagnosed insomnia samples, including individuals with comorbid insomnia and those receiving specialized treatment.

## **Comment 10: Conclusion**

Given that test–retest reliability was below the recommended criterion, the statement regarding the reliability of the Japanese version should be slightly moderated. The conclusion should more accurately reflect that the instrument demonstrated satisfactory internal consistency and construct validity, while acknowledging that temporal stability requires further investigation.

**Response**:

We revised the Conclusion to more accurately reflect the psychometric findings of the present study. Specifically, we moderated the statement regarding the reliability of the Japanese version of the SPAQ by clarifying that the instrument demonstrated acceptable structural validity, internal consistency, construct validity, and measurement invariance, whereas test–retest reliability was not supported. We also explicitly stated that the temporal stability of the SPAQ-J requires further investigation and that score stability should therefore be interpreted with caution.

**Revised**:

**L628~635:** This study demonstrated acceptable structural validity, internal consistency, and construct validity, along with measurement invariance across sex and age, for the two-factor structure of the Japanese version of the SPAQ. These findings suggest that the SPAQ-J can be used as a measure of psychological flexibility in insomnia research, with careful interpretation of each subscale. However, the lower-than-expected test-retest reliability indicates that the temporal stability of the SPAQ-J scores requires further investigation, and score stability should therefore be interpreted with caution. Future research should further investigate temporal stability, sample differences, and the clinical utility of the subscale scores.
